# The International Dental Federation

**Published:** 1901-11-15

**Authors:** E. C. Kirk

**Affiliations:** Philadelphia


### THE INTERNATIONAL DENTAL FEDERATION.

BY DR. E. C. KIRK, OF PHILADELPHIA.

As had been previously announced, the first annual meeting of the International Dental Federation was duly held in Cambridge, England, on Wednesday, August 7th. The occasion was a notable one in many ways, for at no other time in our professional history has a like organization been formed, nor have the circumstances heretofore existed which could bring together so representative an international gathering for a similar object. The idea of forming an international dental federation seems to have had its origin at a meeting of the Copenhagen Dental Society, held in Copenhagen during August, 1894, at which, acting under the suggestion of Dr. George Cunningham, of Cambridge, England, Dr. Haderup, of Copenhagen, presented a paper, "A Proposal for the Foundation of an International Society for Forwarding Dental Education." The proposal was favorably received and discussed at the meeting in question, and a committee of six members from as many countries was appointed, with power to add to their number and to work for the object stated, and within the course of two years to call together an international dental meeting in harmony with the proposal.

At the Third International Congress, held in Paris, in 1900, the idea developed more practical form by a resolution of the Congress creating the "Federation Dentaire Internationale," with its Educational committee and necessary administrative bureau, which gave it a definite working organization and plan.

In the nature of the case the representatives of the several nations concerned in the work of the organization were without delegated authority, hence the work of the organization was unofficial in its national bearings, its results being of importance only in so far as their suggestiveness or inherent value would be of use in improving dental educational ideals or methods. This lack of official character, as the event proved, instead of being a detriment to the work of the organization, was a positive aid, for the free discussion of the educational problem was not at any time hampered by questions of national usage or expediency.

Sixty delegates, representing seventeen countries, assembled in the Physiological Theater of the University Museums at Cambridge, and were welcomed by Sir Michael Foster, M. P., professor of physiology and deputy vice-chancellor of Cambridge University, in an address on dental education which in many respects we regard as the most noteworthy statement which has yet been made of the professional status of the dentist and the principles which should govern his education. Mr. Foster's address was a practical application to dental education of the principles enunciated by Herbert Spencer in his epoch-making essay on education, a clear recognition of the utilitarian character of knowledge and the necessity of shaping education to the uses which the acquired knowledge are to subserve. The problem of the relation of dentistry to medicine was comprehensively stated in his classification of the dentist as a "healer within the limitations of his special sphere of activities," and in his recognition of the necessity for so much of education in the sciences upon which the healing art is based as is necessary to perfect him a as scientific dentist. "While the dental profession doubtless has much to gain in a close alliance with the medical profession, yet one object, and one object only, ought to be the aim of the training of the dentist—*i.e.*, to make him as sure and as efficient a workman as possible."

So frank a recognition of the special character of the training needed by the dentist and the disregard of ancient precedents clearly implied in the ideal of dental education which he has formulated, is at least növel, but, coming as it does from not only a high medical authority, but an authority on medical education as well, is unique, and will carry with it a force which will not fail to make its inherent truth felt in a practical way. The response to Professor Foster's address was made by Dr. Godon, of Paris, president of the Federation, and was a further argument in favor of the special education of the dentist for his professional work. At a later meeting, held in the hall of Trinity College, a general discussion of Sir Michael Foster's address took place, and was participated in by Professor Griffiths, professor of surgery in Cambridge University Medical School; Professor Sims-Woodhead, of the same institution; Sir James Crichton Brown, Dr. J. Leon Williams, Dr. George Cunningham, Dr. Hesse, of Leipzig, Drs. Brophy, Kirk, and others. A singular and surprising unanimity of sentiment in favor of the principles of dental education as set forth in the address of the distinguished chairman was manifested by all the speakers. It was clearly shown that whatever might be the future organic relation of dentistry to medicine, one central conception and ideal must dominate dental education, viz, that the special and definite character of dental practice demands a system of education for the dentist equally special and definite, and that the best educational system is that which will make the best dentist in the broadest meaning of the term.

The social features of the occasion were important not only in themselves, but also because they contributed much toward the harmony and enthusiasm with which the conclusions of the Federation were reached. A luncheon given to the members by Sir Michael Foster in the Combination-room of Trinity College was an event long to be remembered, and a practical recognition upon the part of an ancient and liberal institution of learning of the dignity and importance of our professional craft. At the formal banquet held in the evening at Downing College, Cambridge, an opportunity was given in the post-prandial speeches to still further emphasize the importance of the objects of the Federation, and upon the part of the guests to, in some measure, express their appreciation of the generous hospitality which had been accorded them. At the close of the banquet a lawn *fete* was given to members of the Federation by Dr. Cunningham at his home, Merton Hall, where until a late hour the members enjoyed the opportunities for social intercourse and a further strengthening of the bonds of international confraternity.

We have on previous occasions referred to the great value of the social side of our dental meetings. All have recognized this factor in our local and national gatherings. The need for a better understanding in an international way has long been recognized, but no suitable occasion has heretofore offered outside of the international congresses. The meeting of the Federation was brought about for the avowed purpose of supplying the opportunity for a better and closer international relationship in matters dental, and in that respect it was a success beyond all the expectations of its promoters. It has established a condition of international respect and regard which will certainly strengthen the work of dental education and our professional status the world over by familiarizing each with the national conditions in all other countries, and we trust will finally lead to the evolution of an ideal dental curriculum worthy of universal adoption.

A feature of great contributory interest and importance to the work of the Federation was the annual meeting of the British Dental Association, which was in session for three days in London previous to the meeting of the Federation in Cambridge. It has for many years been a desire of the editor of this journal to attend a meeting of the British Dental Association, for several reasons, but especially to know by practical experience the method of conducting a great national dental meeting in accordance with the English ideal. The experience was in all respects satisfactory, and there was much that would be worthy of adoption at our own meetings in this country. There were the usual features of a large dental meeting—a formal address by the president, scientific essays by members and discussions thereon, clinical demonstrations and exhibits of dental goods. The two most impressive features to the visitor, apart from the excellence of the program, were the system and the good order which prevailed in all departments. It was gratifying to observe that while our English relatives occasionally gave evidences of pugnacity in their debates, especially when a representative from the Emerald Isle manifested a desire to enliven the proceedings by directing the immature British mind along the strait and narrow way, nevertheless at no time did the temperature of debate tend to force it beyond the reasonable limits of parliamentary procedure, and as a consequence the orderly character of the meeting was at no time jeopordized. Our gratification in noting this feature of the meeting grows out of that human sympathy which annimates all individuals with a common feeling, even though it may be the expression of a common weakness. The arrangement of clinics was particularly good ; each operator had a space barred off so that he was free from physical interruption by his audience, yet was close enough for all to see the work or hear its explanation. A placard conveniently placed announced the operation and the name of the operator. The program and the operation of the executive bureau were both excellent, so that the visitor was always able to keep himself fully informed of the progress of events and to find his way easily to the several points of interest.

But beyond all else of interest let us hasten to record in fullest terms of appreciation the cordial reception and generous kindly hospitality which was accorded to their American visitors. We have no doubt that the visitors from other nations will have the same story to tell, but the telling of it we leave to them. It did seem, however, that the cordiality and warmheartedness with which the American delegates were received was special in character, and indicated something deeper than the mere expression of professional brotherhood. It has been more than once said that professional jealousy existed between the dentists of Great Britain and America; let us now and forever forget that any such mistaken idea ever existed. The attitude of the British Dental Association, both as an association and from the point of its individual membership, was a practical and convincing demonstration of the entire absence of any such feeling on their part.

Taken together, the two meetings here referred to have done much to bring about a better understanding among the representatives of the dental profession in all countries, so that one of the greatest obstacles to the scientic and professional advancement of our calling has been removed.

The next meeting of the Federation will be held in Stockholm, where more definite studies of the subject of dental education, it is hoped, will result in still greater advances toward an ideal dental curriculum and a unification of our professional interests.—Editorial in *Cosmos*.

